# Association between intentional behavioral changes and well-being during the COVID-19 pandemic

**DOI:** 10.3389/fpsyg.2023.1201770

**Published:** 2023-07-13

**Authors:** Naoki Konishi, Motohiro Kimura, Yuji Takeda

**Affiliations:** National Institute of Advanced Industrial Science and Technology, Tsukuba, Japan

**Keywords:** psychological well-being, COVID-19, leisure activities, mode of transport, working environment, coping behavior

## Abstract

The enforcement of nationwide lockdowns and social distancing measures severely restricted behavior and led to increases in stress, anxiety, and depression during the COVID-19 Pandemic. However, contrary to expectations, studies show that well-being did not decrease significantly during the Pandemic. The present study examined whether intentional increases in alternative behaviors contributed to maintaining well-being. We predicted an increase in indoor activities as alternatives to outdoor activities and that these behavioral changes contribute to maintaining well-being. Focusing on leisure activities, transport mode, and working environments, we tested these predictions in an online survey of 1,000 participants (*M* = 40.4 years; *SD* = 10.9). The results demonstrated that the decrease in outdoor leisure activities (e.g., traveling and shopping), use of public transportation, and working at office led to a reduction in well-being. It was also demonstrated that the subsequent increase in indoor leisure activities (e.g., exercising at home and online shopping) and use of a private car led to an increase in well-being, which supported our predictions. These results suggest that increasing alternative behaviors can maintain overall well-being during pandemics. These findings highlight the significance of intentional behavioral changes in maintaining well-being during pandemics.

## 1. Introduction

The COVID-19 Pandemic, which started in 2020, has had several effects on people’s daily lives. Several countries have implemented nationwide lockdowns to prevent the virus’s transmission, severely restricting people’s typical behaviors (e.g., van Bavel et al., 2020). The imposition of social distancing measures, including lockdowns and other infection prevention strategies, has restricted behavior and has been detrimental to people’s psychological health and has increased stress, anxiety, and depression (e.g., Khan et al., 2020; Mascherini et al., 2021), potentially leading to a decline in people’s psychological and subjective well-being (e.g., Kuykendall et al., 2018; Iso-Ahola and Baumeister, 2023). However, several studies have suggested that the Pandemic or lockdowns did not significantly affect well-being. For example, van Tilburg et al. (2020) conducted a cross-sectional survey of 1,679 older adults in the Netherlands and reported increased loneliness. Surprisingly, they found no change in happiness levels when comparing happiness in 2019 (before) and 2020 (after the onset of the Pandemic). In addition, Barcellos et al. (2021) assessed changes in several dimensions, including self-rated health status, depressive symptoms, negative emotions, pain, positive affect, and life satisfaction among older adults aged 60–68 years in the United States during the first and second waves of the Pandemic. They reported only a few significant effects of the Pandemic on positive affect indices despite worsening depressive symptoms and negative emotions. O’Connor et al. (2020) studied 3,077 people in the United Kingdom who were intermittently interviewed during March, April, and May 2020 and reported an escalation in suicidal ideation and anxiety levels. Nevertheless, the participants’ well-being showed minimal fluctuations throughout the study. Consistent with these findings, a meta-analysis of 25 longitudinal studies by Prati and Mancini (2021) that included 7,400 participants also shows that positive psychological functions such as well-being and life satisfaction were not significantly reduced during the Pandemic, despite the significant increase in negative emotions such as depression and anxiety.

These findings raise the question as to why the COVID-19 Pandemic failed to have a significant negative impact on well-being. These findings are curious because we would expect restrictions on outdoor activities during the COVID-19 Pandemic to affect psychological health negatively (e.g., Lesser and Nienhuis, 2020; Jackson et al., 2021; Wright et al., 2021; Fernandez et al., 2022; Larson et al., 2022; Quirk et al., 2022). Specific studies had investigated the characteristics of people that maintained good psychological health even when their behavior was restricted. For example, Pouso et al. (2020) reported that Spanish people accessing green or blue natural landscapes at home or in their yards during the lockdown had few depression or anxiety symptoms. Tuason et al. (2021) indicated that individuals who reported higher levels of happiness during the April 2020 lockdown in the United States preferred to spend more time with their family or roommates, engage in outdoor activities, and work from home, whereas those who reported lower levels of happiness spent more time playing games or watching television. Morse et al. (2021) found that people who engaged in creative activities (e.g., arts and crafts) had high levels of psychological health during the COVID-19 Pandemic. These findings indicate that people can maintain or improve their psychological health during activity restrictions, including lockdowns, by engaging in behaviors that avoid infection risks, suggesting that an intentional increase in alternative activities helps mitigate the decline in psychological well-being and sustain psychological health. We hypothesized that people maintained their well-being during the COVID-19 Pandemic through intentional behavioral changes. Specifically, people used modes of transport that avoided infection risks and engaged in home activities to increase their well-being during the Pandemic, rather than going out and maintaining their usual lifestyle despite behavior restrictions, including lockdowns.

Why the COVID-19 Pandemic did not significantly impact well-being is unclear because most studies only investigated the relationship between activities during the lockdown and well-being without directly examining whether people intentionally changed their behavior to maintain and improve their well-being. We designed the current study to evaluate changes in activities that occurred during the COVID-19 Pandemic and current well-being. The study investigated a wide range of activity changes, including leisure activities, not only on non-working days, which previous research has investigated in detail but also modes of transport and the working environment.

### 1.1. Intentional behavioral changes to cope with COVID-19 pandemic

Previous studies examined the relationship between behavior during the Pandemic and well-being without focusing on behavioral changes, particularly the increase in indoor activities (Lesser and Nienhuis, 2020; Tuason et al., 2021). For example, people who preferred to engage in home-based exercise during lockdowns reported higher levels of well-being; however, it was uncertain whether exercising was a habitual behavior before the Pandemic or an intentional behavioral change after the onset of the Pandemic. The present study assessed 16 indoor and 16 outdoor leisure activities and examined the number of behaviors that changed before and after the onset of the COVID-19 Pandemic to address this issue.

In addition, the present study evaluated changes in the modes of transport before and after the onset of the COVID-19 Pandemic. The Pandemic might have affected people’s transport choices because people might have mitigated the adverse effects of the COVID-19 Pandemic on well-being by substituting public transport with high infection risks with alternative modes of transport such as private vehicles, cycling, or walking. The reason for focusing on transportation was that behavioral changes in travel are essential for considering the impact of COVID-19 and well-being. For example, it was found that public transportation use was reduced because contact with others could be frequent and sometimes unavoidable (Tirachini and Cats, 2020).

The COVID-19 Pandemic also changed working environments as remote working became widespread. These transitions might have led to a decrease in face-to-face interactions and an increase in online communication. Changes in the working environment are often unintentional and beyond people’s control. Therefore, they might not be strongly associated with well-being. The present study examined external factors’ influence on the working environment that was beyond the control of individuals and intentional behavioral changes in leisure activities and transport to assess the impact of behavioral changes on well-being comprehensively.

Participants in the present study were residents of Japan. Unlike the United States and European countries that implemented strict lockdowns, the Japanese government only requested travel and activity restrictions from its citizens to limit the spread of COVID-19 but did not mandate them. As a result, Japanese participants’ selection of transport and increased indoor activities were influenced by internal motivations rather than external factors. Therefore, residents of Japan offered an advantage for examining intentional behavioral changes in response to the COVID-19 Pandemic.

### 1.2. Three concepts of well-being

Previous studies that examined well-being during the COVID-19 Pandemic have focused exclusively on a single dimension of well-being. For example, Tuason et al. (2021) focused on eudaimonic well-being, and Pouso et al. (2020) focused on hedonic well-being. The association between one dimension of well-being and a specific activity may be independent of the association between that activity and the other dimension of well-being. For example, Tuason et al. (2021) reported that people with lower eudaimonic well-being preferred engaging in video games and watching television. Research has also suggested that playing video games is associated with hedonic well-being (Johannes et al., 2021). Therefore, it is possible that participants with high hedonic well-being in Tuason, Güss, and Boyd’s study engaged in video games and watching television. As a result, examining multiple dimensions of well-being might be beneficial.

The “hedonic” and “eudaimonic” dimensions are considered essential to well-being traditionally. Hedonic well-being refers to life satisfaction and the ratio of positive to negative emotions, whereas eudaimonic well-being refers to the subjective evaluation of the meaning and purpose of life and experiences. Recently, Oishi et al. (2020a) proposed a third concept of well-being, “psychological richness,” which refers to pursuing diverse and interesting experiences and perspectives. A model of these three dimensions has been proposed as the most appropriate explanation of the “good life” (Oishi and Westgate, 2021). Oishi and his colleagues summarized the characteristics of well-being as follows (Besser and Oishi, 2020). Hedonic well-being involves actively seeking out experiences that bring pleasure and satisfy a person’s desires and striving to secure a stable income and a job that meets a person’s needs for comfort and security. Eudaimonic well-being involves thinking about the experiences that suit a person’s goals and how to best use his or her or their abilities and build a life accordingly. Psychological richness involves experiencing new situations without worrying about stability or comfort and following a person’s curiosity in real life or through imagination. Hence, the effects of different behavioral changes might differ depending on the specific type of well-being. In the present study, we comprehensively assessed hedonic well-being, eudaimonic well-being, and psychological richness to examine the relationship between these three dimensions of well-being and behavioral changes.

### 1.3. Purpose and hypotheses

The purpose of the present study was to examine the hypothesis that well-being could be maintained if people adopted an alternative lifestyle under the restriction by COVID-19. Previous studies have focused only on the relationships between the “current” lifestyle and well-being. Beyond the previous studies, in the present study, we focused on the relationships between “behavioral changes” due to the COVID-19 pandemic and three dimensions of well-being.

More specifically, the present study investigated whether increases in alternative activities due to behavioral restrictions during the Pandemic resulted in maintaining or improving well-being by examining the relationship between behavioral changes before and after the onset of the Pandemic on well-being. We hypothesized that the association between behavioral changes and well-being varies according to the type of activity. We predicted a decrease in outdoor recreational activities and a corresponding increase in indoor activities due to the Pandemic. We also expected that the decrease in outdoor activities would lead to a decrease in well-being, whereas the increase in indoor activities would lead to an increase in well-being, ultimately contributing to maintaining overall well-being.

We also expected that the use of public transport, a high infection-risk mode of transport, would decrease, whereas the use of private vehicles, cycling, and walking would increase as an alternative to public transport might increase during the COVID-19 Pandemic. The decrease in using public transport might lead to a decrease in well-being. However, we also expected that an increase in using private vehicles, cycling, and walking as a substitute for public transport would result in maintaining or improving well-being.

Finally, we expected working from home to increase following the COVID-19 Pandemic. The nature of a job and a company’s policy, rather than the workers’ choice, frequently determines the working environment choice. Therefore, if well-being were maintained or improved through intentional (but not unintentional) behavioral changes, we expected that an increase in working from home would affect the well-being of people who could freely choose their working environments but those who could not choose.

Different dimensions of well-being are likely to be influenced by different motivational factors (Oishi et al., 2020b; Bojanowska et al., 2021), resulting in different relationships between different well-being dimensions and outdoor and indoor activities. Hedonic well-being might be more strongly associated with outdoor activities (including transport) than indoor activities because hedonic well-being is enhanced by social activities (e.g., parties, exercising, and shopping; Diener, 1984). On the other hand, eudaimonic well-being does not necessarily require pleasurable outcomes, as it emphasizes the experiences of meaning and purpose (Waterman, 1993). Therefore, eudaimonic well-being might be maintained or improved even by doing indoor activities. As a result, we expected that eudaimonic well-being would be associated not only with outdoor activities but also with indoor activities. Finally, psychological richness does not focus on outcomes and purpose but requires “new” experiences (Besser and Oishi, 2020). Therefore, we expected psychological richness to be associated with outdoor and indoor activities. It might be possible that psychological richness is more closely associated with indoor than outdoor activities during the Pandemic because the Pandemic restricted new outdoor activities.

## 2. Method

### 2.1. Participants

We conducted a nationwide Internet survey through a survey company (MyVoice Communications, Inc) in November 2022. The survey company sent an email invitation to potential candidates of the survey. Individuals who expressed their willingness voluntarily accessed the website to complete the questionnaire on a first-come, first-served basis. Participants in the present study were collected with the constraint that the proportions of combinations of gender (male and female) and age (20, 30, 40, 50, and 60s) were equal. All participants were residents in Japan (*N* = 1,000, 500 men and 500 women; Mean age, 40.4 years; *SD* = 10.7, Age range, 20–60 years). Participants who gave the same answer to all single questions or who finished answering questions in an extremely short period were excluded. After the exclusion, a total of 1,000 responses were obtained. Because Tuason et al. (2021) used a sample size of 977 participants, we used a sample size of 1,000 to maintain similar robustness of the sample.

The survey was conducted in early October 2022; the number of new cases per 100,000 population was about 30–40. In Japan, COVID-19 control measures did not vary significantly from region to region. Only recommendations were made regarding home and travel restrictions; requests from the government were issued to “refrain from going out unless necessary” and “refrain from holding events.” In fact, policy response in Japan was relatively weak compared to other countries (Hale et al., 2021).

### 2.2. Procedure

We instructed the participants to recall their behaviors in 2019 and compare them with their current behaviors to assess behavioral changes caused by the Pandemic. We provided the participants with lists of significant events in 2019 and 2021 to facilitate memory retrieval. Then, we asked them to recall and write down an event that happened to them in 2019. After being reminded often of 2019 and 2021, we also asked them to report how their current leisure activities, modes of transport, and work environments have changed compared to 2019. The reason for including the recall method was that the present study aimed to selectively focus on “behavioral changes” before and after COVID-19 quantitatively. To confirm the validity of the behavioral change measure from 2019, we also measured participants’ lifestyle changes due to COVID-19. Next, we asked participants to respond to the COVID-19 Fear Scale and the COVID-19 Coping Behavior Scale (Wakashima et al., 2020) to determine whether the change in behavior from 2019 was related to attitudes toward COVID-19. We measured participants’ well-being using a scale corresponding to the 3 dimensions. Finally, the participants also provided their demographic information, including gender, age, income, and marital status, which we used as control variables in the present study.

### 2.3. Materials

#### 2.3.1. Behavioral changes due to COVID-19 pandemic

The present study used a comprehensive battery of measures to recall 3 types of behavioral changes due to COVID-19 Pandemic. First, we developed a 32-item questionnaire based on previous research by Densley et al. (2013), and Tuason et al. (2021) for assessing outdoor (16 items) and indoor (16 items) activities. The outdoor activities included items such as “visiting libraries and bookstores,” “exercising, walking, playing sports outdoors,” and “going to the movies.” The indoor activity category included items such as “reading literature (novels, poetry, newspapers, news, magazines),” “exercising, stretching, doing yoga at home,” and “using movie streaming services (e.g., Netflix, Amazon Prime, Hulu).”

Second, we also assessed changes in transport modes that the participants used by requesting them to indicate how their use of six modes of transport, including “private vehicles (driver),” “private vehicles (passenger),” “cycling,” “motorcycles,” “busses (transit busses, community busses),” “taxis,” “trains,” and “walking” have changed compared to 2019. The participants separately responded about the transport they used on weekdays and weekends. Because of the high correlation between them (*r*s = 0.76–0.86), we analyzed the average of transport used on weekdays and weekends.

Third, we asked the participants to report the frequencies of encountering four aspects of their working environments, including working from home, workations, face-to-face communications, and online communications, and to indicate how these frequencies have changed compared to 2019 to assess changes in the participants’ working environments. Participants responded using a 5-point scale ranging from 1 (*Greatly decreased or Use much less*) to 5 (*Greatly increased or Use much more use*). We also gave the participants the option of selecting “I did not perform this activity (used specific modes of transport, experience this working environment) in 2019 or perform it currently.” Additionally, the work-from-home condition is often unintentional and beyond the control of individuals. Therefore, we assessed the degree of autonomy that participants had when deciding to work from home using a 5-point Likert scale ranging from 1 (*No autonomy at all*) to 5 (High degree of autonomy).

We also assessed the participants’ lifestyle changes caused by the Pandemic to validate the composite behavioral changes calculated by combining the above items. We requested the participants to indicate the extent to which the following eight lifestyle variables had changed compared to before the outbreak: going on vacations, staying home on vacations, working outside the home, working inside the home, commuting using public transport, using public transport for leisure, commuting by walking, and walking for leisure. Participants responded using a 5-point Likert scale ranging between 1 (*Greatly decreased*) to 5 (*Greatly increased*).

#### 2.3.2. Assessing COVID-19 fear and attitudes

We could not be sure that the Pandemic caused any of the behavioral changes we observed after 2019 using the above measures. Therefore, we used the Fear of COVID-19 Scale and the COVID-19 Coping Behavior Scale developed by Wakashima et al. (2020) to confirm the relationship between behavioral changes and the fear and attitudes about COVID-19. The Japanese version of the Fear of COVID-19 Scale consists of 7 items (*α* = 0.92) assessing a person’s fear of the virus. Participants rate their agreement with statements such as “COVID-19 is most frightening” and “I cannot calm down when I think about COVID-19,” using a 5-point Likert scale ranging from 1 (*Strongly disagree*) to 5 (*Strongly agree*). The COVID-19 Coping Behavior Scale consists of 13 items assessing a person’s response to the virus. This scale includes 8 items (*α* = 0.81) assessing the care taken in daily life (e.g., “Avoided places with large crowds”), 2 items (*α* = 0.84) assessing stockpiling (e.g., “Purchased commodities in larger quantities than usual”), and 3 items (*α* = 0.67) assessing health monitoring (e.g., “Monitored health condition more carefully than before”). Participants rated their agreement with each item using a 7-point Likert scale ranging from 1 (*Not at all*) to 7 (*Quite a lot*). The alpha coefficients in the present study for these subscales were comparable to those in Wakashima et al. (2020), which confirmed the adequate internal validity.

#### 2.3.3. Assessing well-being

We assessed the participants’ well-being using several established scales. Hedonic well-being was assessed using the Satisfaction with Life Scale (SWLS, Diener et al., 1985; *α* = 0.91). The participants rated the five SWLS items using a scale ranging from 1 (*Strongly disagree*) to 7 (*Strongly agree*). We assessed eudaimonic well-being using the Meaning in Life Questionnaire (Steger et al., 2006), which consists of 10 items: 5 items assessing the presence of meaning (MLQ-presence; *α* = 0.85) and 5 items assessing the search for meaning (MLQ-search; *α* = 0.92). Participants rated each item on a scale from ranging from 1 (*Strongly disagree*) to 7 (*Strongly agree*). We used the Psychologically Rich Life Questionnaire developed by Oishi et al. (2019), which comprises 17 items (*α* = 0.93) to assess psychological richness. Participants rated each item on a scale ranging from 1 (*Strongly disagree*) to 7 (*Strongly agree*). Participants also responded to the Japanese version of the Scale of Positive and Negative Experience (SPANE, Diener et al., 2010; Sumi, 2014). The Japanese iteration of the Scale of Positive and Negative Experience (SPANE, Diener et al., 2010) was employed to quantify participants’ positive affect related to hedonic well-being. The scale consists of 6 items assessing positive emotion (*α* = 0.93) and 6 items assessing negative emotion (*α* = 0.89). Because SWLS reflects the state of hedonic well-being more directly, SPANE were not included in the analyses. The alpha coefficients in the present study for these scales were comparable to those in previous studies (Diener et al., 1985; Steger et al., 2006; Oishi et al., 2019), which confirmed the adequate internal validity. Because the correlations among the three dimensions of well-being were considerably high (SWLS and MLQ-presence; *r* = 0.58, MLQ-presence and Psychologically richness; *r* = 0.69, Psychologically richness and SWLS; *r* = 0.63), each of them will be analyzed independently for avoiding multicollinearity problems.

### 2.4. Data analyses

Statistical analyses were conducted using the psych package (Revelle, 2020), in the R statistical language (Version 3.6.3; R Core Team, 2020).

The analyses of the present study were performed as follows: (1) behavioral changes in comparison between 2019 (before) and 2020 (after the onset of the Pandemic) were examined (see Section 3.1). (2) specific behavioral-change items related to COVID-19 were selected and behavioral-change variables were constructed (see Section 3.2). (3) Validation of the constructed behavioral-change variables was performed (see Section 3.3). (4) The relationships between these variables and the three dimensions of well-being were examined independently by conducting multiple linear regression analyses (see Section 3.4).

## 3. Results

### 3.1. Activity changes during the pandemic

We transformed the numerical values of the 5-point evaluation scales, e.g., 1 (*Greatly decreased*) to 5 (*Greatly increased*) assessing behavioral changes in leisure activities, transport modes, and working environments into a scale of-2 (*Greatly decreased*) to 2 (*Greatly increased*), such that a negative numerical value denoted a decrease in behavior. In contrast, a positive value denoted an increase in behavior compared to 2019. Table 1 shows the descriptive statistics of behavioral changes in leisure activities, transport modes, and working environments. We treated participants who reported they had not experienced a behavior (e.g., responding “I did not perform this activity in 2019 or perform it currently”) as missing values.

**Table 1:** Sample size and mean values of behavioral changes and their correlation with coping behaviors in response to COVID-19.

		*N*	*M*	*r (correlation with coping behaviors)*	
Leisure activity (outdoor activity; 1–16, indoor activity; 17–32)					
**1**	Visiting libraries or bookstores	696	−0.40	−0.11	*
**2**	Participating in artistic events (e.g., concerts, art exhibitions, performances),	561	−0.73	−0.24	**
**3**	Attending club or group meetings, practices, workshops, or career-related fairs	393	−0.65	−0.26	**
4	Exercising, walking, or playing sports outdoors	695	−0.27	−0.03	
**5**	Traveling (e.g., fruit-picking, hiking, hot springs)	811	−0.83	−0.20	**
**6**	Watching sports at stadiums (e.g., baseball, soccer)	451	−0.72	−0.14	*
**7**	Shopping	952	−0.43	−0.15	**
**8**	Spending time with friends or going on dates	794	−0.92	−0.18	**
**9**	Attending theme parks or game events	504	−0.73	−0.20	**
10	Participating in community or neighborhood events	410	−0.62	−0.08	
11	Volunteering	327	−0.37	−0.01	
**12**	Visiting cafes or restaurants	866	−0.67	−0.23	**
**13**	Going to bars or clubs	722	−1.10	−0.23	**
**14**	Going to the movies	695	−0.71	−0.19	**
15	Taking lessons	340	−0.34	0.07	
16	Going to sports gyms or fitness clubs	375	−0.38	−0.09	
**17**	Reading literature (e.g., novels, poetry, newspapers, news, magazines)	732	−0.03	0.13	*
18	Engaging in artistic activities at home (e.g., painting, Playing music, crafting)	416	−0.19	0.11	
19	Participating in online workshops or fairs	334	0.02	0.15	
**20**	Exercising, stretching, or yoga at home	586	0.11	0.13	*
21	Engaging in online travel or tours	268	−0.31	0.13	
**22**	Watching sports on TV or the internet (e.g., baseball, soccer)	642	−0.06	0.17	**
**23**	Online shopping	955	0.36	0.23	**
**24**	Making online calls or dates	424	0.19	0.18	**
**25**	Playing video games (e.g., Switch, PlayStation, PC) or social games	581	0.11	0.14	**
26	Making donations	421	−0.13	0.10	
**27**	Ordering food delivery or using Uber Eats	461	0.19	0.19	**
28	Having online or small-scale drinking parties at home	399	−0.16	0.09	
**29**	Using video streaming services (e.g., Netflix, Amazon Prime, Hulu)	578	0.43	0.25	**
**30**	Taking online classes or lessons	336	−0.09	0.19	**
**31**	Home gardening or engaging in gardening	396	−0.02	0.21	**
**32**	Relaxing at home	952	0.48	0.17	**
**Transport**					
**1**	Private vehicles (driver)	734	0.03	0.16	**
2	Private vehicles (passenger)	613	−0.22	0.07	
**3**	Cycling	576	−0.05	0.15	**
4	Using bikes	279	−0.20	0.13	
**5**	Using busses (transit bus, community bus)	600	−0.39	−0.11	*
6	Using taxis	468	−0.43	−0.06	
**7**	Using trains	807	−0.46	−0.13	**
**8**	Walking	886	0.04	0.12	**
Working environment					
**1**	Work from home	490	0.73	0.13	*
**2**	Workation	239	−0.22	0.18	*
**3**	Face-to-face communication	821	−0.65	−0.16	**
**4**	Online communication.	585	0.57	0.18	**

*N* represents sample size excluding missing value. *M* and *r* represent mean and correlation coefficients with care taken in daily life for COVID-19, respectively. Bold numbers are behavioral-change items related to COVID-19 that were included in the analysis; *** indicates *p* < 0.001; ** indicates *p* < 0.01; * indicates *p* < 0.05.

Table 1 shows that activities involving going out and contacting others decreased during the Pandemic, whereas activities that could be done at home and avoided contact with others increased. All 16 outdoor leisure activities decreased, such as going to bars and clubs (*M* = −1.10) and traveling (*M* = −0.83). In contrast, 8 of the 16 indoor leisure activities increased, such as exercising, stretching, doing yoga at home (*M* = 0.11), and using video streaming services (*M* = 0.43). Concomitantly, using public transport, such as trains (*M* = −0.46) and busses (*M* = −0.39), decreased. In contrast, using transport that avoided contact with others, such as private vehicles (driver) (*M* = 0.03) and walking (*M* = 0.04), increased. Furthermore, working from home (*M* = 0.73) increased in 2022 compared to 2019, with a corresponding decrease in face-to-face communication during work (*M* = −0.65).

### 3.2. Association between behavioral changes and COVID-19

The present study was designed to examine behavioral changes caused by the COVID-19 Pandemic. Therefore, we selected items significantly related to attitudes about COVID-19 in the following analysis. We selected these items by calculating the correlation coefficients between behavioral change items and the care taken in daily life about COVID-19 assessed by coping behavior scale. Table 1 displays the correlation coefficients. The items with bold numbers were included in the analysis.

We observed a significant negative correlation in 11 of the 16 outdoor leisure activities; participants who were more cautious about COVID-19 tended to reduce their engagement in these outdoor activities. In contrast, 11 of the 16 indoor activities showed a significant positive correlation; participants who were more cautious about COVID-19 tended to increase their engagement in these indoor activities. We calculated the means of 11 outdoor (*α* = 0.90) and 11 indoor activities (*α* = 0.86) as the outdoor activity and the indoor activity variables.

The use of busses and trains was negatively correlated with caution about COVID-19, indicating that more cautious participants tended to use less public transport. We combined these two items (*r* = 0.58) as the public transport variable. Positive correlations were observed in using private vehicles (driver), bicycles, and walking, indicating that participants who were more cautious about COVID-19 tended to increase using these modes of transport. However, the scale’s internal consistency was insufficient (*α* = 0.40) to merge these three items (i.e., private vehicles (driver), bicycles, and walking). Therefore, we analyzed them separately as non-contact transport variables.

Face-to-face communication in the working environment was negatively correlated with caution about COVID-19, indicating that participants more cautious about COVID-19 tended to decrease working in offices. We analyzed this item as the work-in-offices variable. In contrast, working from home, workations, and online communications were positively correlated with caution about COVID-19, indicating that participants who were more cautious about COVID-19 tended to increase these working styles. We combined these three items (*α* = 0.74) as the homeworking variable.

### 3.3. Validity of behavioral changes due to COVID-19

To validate the composite variables consisting of outdoor activities, indoor activities, public transport, and homeworking, we conducted a correlational analysis between these composite variables and participants’ lifestyle changes due to the COVID-19 Pandemic. The results indicated that outdoor activities were positively correlated with going out on vacations [*r*(987) = 0.36, *p* <0.001]. Moreover, indoor activities were positively correlated with staying at home during vacation [*r*(995) = 0.35, *p* <0.001]. Also, public transport was positively correlated with using public transport [commuting; *r*(821) = 0.35, *p* < 0.001, leisure; *r*(821) = 0.46, *p* <0.001]. Finally, homeworking was positively correlated with working inside the home [*r*(653) = 0.40, *p* < 0.001]. These results suggest that each composite variable was valid.

### 3.4. Behavioral changes due to COVID-19 and well-being

We examined the relationship between behavioral changes due to the COVID-19 Pandemic and well-being by conducting multiple linear regression analyses. Table 2 shows that means, standard deviations, and correlations for outcome variables (hedonic well-being, eudaimonic well-being, and psychological richness) and predictors (leisure activities, transport modes, and working environments). The moderate correlations among the target predictors (e.g., indoor activity and homeworking: *r* = 0.45, outdoor activity and public transport: *r* = 0.41) were observed, which could cause multicollinearity problems. In addition, the numbers of the variables seem too large to include in a single multiple regression model. Therefore, three types of target predictors were analyzed independently.

**Table 2:** Means, standard deviations, and correlations for outcome variables and predictors.

Variable		*M*	*SD*	1	2	3	4	5	6	7	8	9	10	11
1	Hedonic well-being (life satisfaction)	3.63	1.32											
2	Eudaimonic well-being (meaning in life)	3.71	1.13	0.58^**^										
3	Psychological richness	3.92	0.99	0.63^**^	0.69^**^									
4	Indoor activity	0.23	0.59	0.07^**^	0.10^**^	0.11^**^								
5	Outdoor activity	−0.70	0.70	0.09^**^	0.08^**^	0.05	−0.05							
6	Private vehicles (driver)	0.03	0.79	0.13^**^	0.13^**^	0.09^*^	0.21^**^	0.06						
7	Cycling	−0.05	0.90	0.01	0.05	−0.01	0.26^**^	0.04	0.13^**^					
8	Walking	0.04	0.72	0.05	0.07^*^	0.08^*^	0.22^**^	0.10^**^	0.11^**^	0.30^**^				
9	Public transport (busses, trains)	−0.45	0.85	0.07^*^	0.11^**^	0.09^**^	0.01	0.41^**^	−0.08	0.13^**^	0.27^**^			
10	Homeworking	0.58	0.98	0.02	−0.05	0.02	0.45^**^	−0.10^*^	0.19^**^	0.24^**^	0.16^**^	−0.08^*^		
11	Work at office	−0.65	0.91	0.04	0.03	−0.04	−0.10^*^	0.36^**^	0.02	0.05	0.10^**^	0.31^**^	−0.34^**^	
12	Control of homeworking	2.18	1.42	0.16^**^	0.13^**^	0.16^**^	−0.00	0.03	0.02	−0.01	0.05	−0.09^**^	0.25^**^	−0.23^**^

*M* and *SD* are used to represent mean and standard deviation, respectively. “Indoor activity” indicates an increase or decrease in leisure activity at home, while “outdoor activity” indicates an increase or decrease in leisure activity that involves going out. “Private vehicles (driver),” “cycling,” and “walking” represent increases or decreases in private vehicle travel, cycling, and walking, respectively, while “public transport (busses, trains)” represents the increase or decrease in travel by public transport. “Homeworking” indicates an increase or decrease in work at home, while “work at office” indicates an increase or decrease in work outside of the home; ** indicates *p* < 0.01; * indicates *p* < 0.05.

Table 3 presents the results of the analyses for testing leisure activities (outdoor activities and indoor activities) as the independent variable predicting well-being analyses with gender, age, marital status, and income as the control variables. The results showed that participation in indoor leisure activities was positively associated with hedonic well-being [*β* = 0.10, *t*(987) = 2.37, *p* = 0.017], eudaimonic well-being [*β* = 0.12, *t*(987) = 3.50, *p* < 0.001], and psychological richness [*β* = 0.11, *t*(987) = 3.42, *p* < 0.001]. Moreover, outdoor leisure activities were correlated with hedonic well-being [*β* = 0.16, *t*(987) = 3.79, *p* < 0.001], eudaimonic well-being [*β* = 0.12, *t*(987) = 3.20, *p* = 0.001], and psychological richness [*β* = 0.09, *t*(987) = 2.87, *p* = 0.004].

**Table 3:** Results of multiple regression analysis examining the effect of leisure activity on well-being dimensions (hedonic well-being, eudaimonic well-being, psychological richness).

	*β*	*t*	95% CI
Hedonic well-being			
(Intercept)	3.93***	49.43	[3.87, 3.98]
Gender (0; male, 1; female)	−0.16	−1.96	[−0.22, −0.11]
Age	−0.16***	−3.81	[−0.19, −0.13]
Marriage (0; unmarried. 1; married)	−0.39***	−4.29	[−0.46, −0.33]
Income	0.28***	6.40	[0.25, 0.31]
Indoor activity	0.10*	2.37	[0.07, 0.12]
Outdoor activity	0.16***	3.79	[0.13, 0.19]
Eudaimonic well-being			
(Intercept)	3.82***	55.43	[3.77, 3.87]
Gender (0; male, 1; female)	0.07	0.97	[0.02, 0.12]
Age	−0.12**	−3.16	[−0.14, −0.09]
Marriage (0; unmarried. 1; married)	−0.27***	−3.36	[−0.32, −0.21]
Income	0.21***	5.38	[0.18, 0.23]
Indoor activity	0.12***	3.51	[0.1, 0.15]
Outdoor activity	0.12**	3.20	[0.09, 0.14]
Psychological richness			
(Intercept)	4.13***	68.04	[4.09, 4.17]
Gender (0; male, 1; female)	−0.24***	−3.91	[−0.29, −0.2]
Age	−0.03	−0.80	[−0.05, 0]
Marriage (0; unmarried. 1; married)	−0.15*	−2.19	[−0.2, −0.11]
Income	0.18***	5.23	[0.15, 0.2]
Indoor activity	0.11***	3.42	[0.08, 0.13]
Outdoor activity	0.09**	2.87	[0.07, 0.11]

β and CI indicate the standardized regression weights and the confidence interval, respectively. “Indoor activity” indicates an increase or decrease in leisure activity at home, while “outdoor activity” indicates an increase or decrease in leisure activity that involves going out. “Hedonic well-being” and “Eudaimonic well-being” were measured by the Satisfaction with Life Scale and the Meaning in Life Questionnaire, respectively; *** indicates *p* < 0.001; ** indicates *p* < 0.01; * indicates *p* < 0.05.

Table 4 presents the results of multiple linear regression analyses that tested transport modes [public transport, private vehicles (driver), cycling, and walking] as independent variables predicting the well-being dimensions. The results indicated that using public transport was positively correlated with hedonic well-being [*β* = 0.18, *t*(418) = 2.84, *p* = 0.004], eudaimonic well-being [*β* = 0.15, *t*(418) = 2.89, *p* = 0.004], and psychological richness [*β* = 0.17, *t*(418) = 3.82, *p* < 0.001]. Moreover, using private vehicles (driver) was associated with hedonic well-being [*β* = 0.21, *t*(418) = 3.24, *p* = 0.001], eudaimonic well-being [*β* = 0.18, *t*(418) = 3.78, *p* < 0.001], and psychological richness [*β* = 0.09, *t*(418) = 2.12, *p* = 0.03]. Conversely, cycling was negatively associated with psychological richness [*β* = −0.14, *t*(418) = −2.96, *p* = 0.003]. However, walking was not significant association with any well-being measure.

**Table 4:** Results of multiple regression analysis examining the effect of modes of transport on well-being dimensions (hedonic well-being, eudaimonic well-being, psychological richness).

	*β*	*t*	95% CI
Hedonic well-being			
(Intercept)	4.03***	34.85	[3.95, 4.11]
Gender (0; male, 1; female)	−0.05	−0.39	[−0.13, 0.03]
Age	−0.14*	−2.16	[−0.18, −0.09]
Marriage (0; unmarried. 1; married)	−0.41**	−2.94	[−0.5, −0.31]
Income	0.20**	2.97	[0.15, 0.25]
Private vehicles (driver)	0.20**	3.24	[0.15, 0.24]
Cycling	−0.01	−0.14	[−0.05, 0.04]
Walking	−0.02	−0.36	[−0.07, 0.02]
Public transport (busses, trains)	0.18**	2.84	[0.14, 0.22]
Eudaimonic well-being			
(Intercept)	3.91***	42.35	[3.85, 3.97]
Gender (0; male, 1; female)	0.17	1.75	[0.1, 0.23]
Age	−0.11*	−2.10	[−0.14, −0.07]
Marriage (0; unmarried. 1; married)	−0.21	−1.88	[−0.28, −0.13]
Income	0.09	1.71	[0.06, 0.13]
Private vehicles (driver)	0.18***	3.78	[0.15, 0.21]
Cycling	−0.05	−1.01	[−0.09, −0.02]
Walking	0.01	0.15	[−0.03, 0.04]
Public transport (busses, trains)	0.15**	2.89	[0.11, 0.18]
Psychological richness			
(Intercept)	4.14**	49.31	[4.08, 4.2]
Gender (0; male, 1; female)	−0.14	−1.56	[−0.19, −0.08]
Age	−0.03	−0.71	[−0.06, 0]
Marriage (0; unmarried. 1; married)	−0.06	−0.63	[−0.13, 0]
Income	0.14**	2.95	[0.11, 0.18]
Private vehicles (driver)	0.09*	2.12	[0.06, 0.12]
Cycling	−0.14**	−2.96	[−0.17, −0.11]
Walking	0.07	1.43	[0.04, 0.1]
Public transport (busses, trains)	0.17**	3.83	[0.14, 0.21]

β and CI indicate the standardized regression weights and the confidence interval, respectively. “Private vehicles (driver),” “cycling,” and “walking” represent increases or decreases in private vehicle travel, cycling, and walking, respectively, while “public transport (busses, trains)” represents the increase or decrease in travel by public transport. “Hedonic well-being” and “Eudaimonic well-being” were measured by the Satisfaction with Life Scale and the Meaning in Life Questionnaire, respectively; *** indicates *p* < 0.001; ** indicates *p* < 0.01; * indicates *p* < 0.05.

Table 5 presents the results of multiple linear regression analyses testing working environments (work-in-offices and homeworking) as independent variables predicting well-being dimensions with gender, age, marital status, income, and the subjective level of controlling homeworking as the control variables. Results indicate that work-in-offices was positively correlated with hedonic [*β* = 0.14, *t*(617) = 2.61, *p* = 0.009] and eudaimonic well-being [*β* = 0.09, *t*(617) = 2.00, *p* = 0.046]. However, psychological richness was not correlated [*β* = 0.04, *t*(617) = 0.93, *p* = 0.352]. Homeworking did not correlate significantly with any measure of well-being examined in the present study. Results also showed that subjective control of work from home was positively correlated with hedonic well-being [*β* = 0.18, *t*(617) = 3.44, *p* < 0.001] and psychological richness [*β* = 0.08, *t*(617) = 1.97, *p* = 0.049].

**Table 5:** Results of multiple regression analysis examining the effect of working environment on well-being dimensions (hedonic well-being, eudaimonic well-being, psychological richness).

	*β*	*t*	95% CI
Hedonic well-being			
(Intercept)	4.06**	43.07	[4.00, 4.12]
Gender (0; male, 1; female)	−0.16	−1.57	[−0.22, −0.09]
Age	−0.18***	−3.52	[−0.22, −0.15]
Marriage (0; unmarried. 1; married)	−0.37***	−3.34	[−0.44, −0.30]
Income	0.18***	3.22	[0.14, 0.21]
Control of homeworking	0.18**	3.44	[0.14, 0.21]
Homeworking	0.05	0.94	[0.01, 0.09]
Work at office	0.14**	2.61	[0.10, 0.17]
Eudaimonic well-being			
(Intercept)	3.89***	47.68	[3.84, 3.95]
Gender (0; male, 1; female)	0.12	1.41	[0.06, 0.18]
Age	−0.07	−1.55	[−0.10, −0.04]
Marriage (0; unmarried. 1; married)	−0.14	−1.49	[−0.21, −0.08]
Income	0.16**	3.30	[0.12, 0.19]
Control of homeworking	0.09	1.94	[0.06, 0.12]
Homeworking	−0.03	−0.64	[−0.06, 0.00]
Work at office	0.09*	2.00	[0.06, 0.12]
Psychological richness			
(Intercept)	4.21***	57.45	[4.16, 4.26]
Gender (0; male, 1; female)	−0.17*	−2.24	[−0.23, −0.12]
Age	−0.02	−0.48	[−0.05, 0.01]
Marriage (0; unmarried. 1; married)	−0.08	−0.93	[−0.14, −0.02]
Income	0.13**	2.95	[0.1, 0.15]
Control of homeworking	0.08*	1.97	[0.05, 0.11]
Homeworking	0.03	0.81	[0.01, 0.06]
Work at office	0.04	0.93	[0.01, 0.07]

β and CI indicate the standardized regression weights and the confidence interval, respectively. “Homeworking” indicates an increase or decrease in work at home, while “work at office” indicates an increase or decrease in work outside of the home. “Hedonic well-being” and “Eudaimonic well-being” were measured by the Satisfaction with Life Scale and the Meaning in Life Questionnaire, respectively; *** indicates *p* < 0.001; ** indicates *p* < 0.01; * indicates *p* < 0.05.

To explore the comprehensive relationships among three types of target predictors (leisure activities, transport modes, and working environments), we also conducted multiple regression analyses selectively including significant variables observed in the separately conducted analyses. The results showed that most variables were significantly associated with each well-being, although some variables had weaker associations (see Supplementary Table S1).

## 4. Discussion

### 4.1. Behavioral changes and well-being

The present study examined if behavioral changes caused by the COVID-19 pandemic were related to maintaining and improving well-being. We predicted a decrease in outdoor and an increase in indoor leisure activities resulting from the Pandemic. The results supported this prediction. We also predicted that a decrease in outdoor activities would lead to a decrease in well-being, whereas an increase in indoor activities would lead to an increase in well-being. The results supported this prediction by showing that changes in outdoor activities were positively associated with well-being, such that people who decreased their outdoor also decreased their well-being. The results also showed that changes in indoor activities were positively associated with well-being, such that people who increased their indoor activities also increased their well-being. Therefore, the present study supported the contention that despite decreasing outdoor activities during the Pandemic, people maintained their overall well-being by increasing indoor activities. Previous studies have reported that well-being was unchanged during the Pandemic, whereas anxiety and depression increased (e.g., O’Connor et al., 2020; Sibley et al., 2020; van Tilburg et al., 2020; Barcellos et al., 2021; Prati and Mancini, 2021). These studies explained how well-being was maintained during the Pandemic using concepts of resilience or coping behavior, which they did not specifically examine. Coping with the COVID-19 pandemic was demonstrated in the present study, which indicated that reducing outdoor activities led to a decrease in well-being, whereas increasing indoor activities as a coping behavior led to an increase and maintaining well-being. These findings suggest the crucial role of alternative indoor activities as coping behaviors for maintaining well-being. People who could not make intentional behavioral changes (i.e., who did not increase indoor activities) experienced lower well-being due to the pandemic.

We also predicted that the use of public transport would decrease, whereas using private vehicles, cycling, and walking would increase as an alternative to public transport during the COVID-19 pandemic. The results showed that using public transport, using private vehicles (passenger), and cycling decreased, and using private vehicles (driver) and walking increased during the Pandemic. Additionally, we predicted that a decrease in using public transport would lead to a decrease in well-being, whereas an increase in using private vehicles, cycling, and walking would lead to maintaining or improving well-being. The results suggested that changes in using public transport positively correlated with well-being, such that avoiding busses and trains to reduce the risk of infection led to a decrease in well-being. In addition, using private vehicles (driver) was positively associated with well-being. However, there was no correlation between cycling/walking and well-being, suggesting that individuals who drove private vehicles as an alternative to public transport could maintain or improve their well-being. Furthermore, the lack of an association between cycling/walking and well-being might be because they were not viable substitutes for public transport. The distance traveled by cycling or walking is limited compared to private vehicles. As a result, increasing cycling or walking had no positive effect on well-being. Therefore, only private vehicles could be a viable alternative to public transport. We also found a negative association between cycling and psychological richness, suggesting that individuals who increased or decreased their cycling decreased or increased psychological richness. The reasons for this relationship remain unclear, but it was specific to psychological richness because we found no significant correlation with other well-being dimensions and cycling. We suggest that future studies investigate this issue further.

Finally, we predicted that working from home would increase during the COVID-19 pandemic. The study indicated that working from home increased during the Pandemic. Unexpectedly, the decrease in face-to-face communication at work was associated with decreased well-being. Moreover, working from home was not associated with well-being. These findings suggest that interpersonal communication might be crucial for improving well-being, even at work. The Japanese work-life balance favors work (Japan spent the 5th most time at work out of 41 countries: OECD, 2020). Therefore, communication with colleagues at work might be essential for maintaining well-being in Japan because social interactions and connections are essential for well-being (e.g., Diener, 1984; Sandstrom and Dunn, 2014; Holt-Lunstad et al., 2015). Therefore, it is plausible that the positive association between face-to-face communication at work and well-being was not solely caused by working in an office but rather a consequence of reduced communication. In addition, we found a positive relationship between the degree of autonomy to decide on the own work environment and well-being, regardless of whether a person worked from home or an office, suggesting that behavioral autonomy is essential for improving well-being, regardless of the working environment.

We also predicted different relationships between different types of well-being and behavioral change, such that hedonic well-being is more strongly associated with outdoor activities, including transport, than indoor activities. However, the results did not differentiate associations among the three well-being dimensions. The regression coefficients might imply that outdoor activities and transport were more closely related to hedonic well-being than other dimensions, although this contention is speculative. We expected that eudaimonic well-being is associated with outdoor and indoor activities. The regression coefficients showed that indoor activities were more closely associated with eudaimonic well-being than other activities. We also predicted that psychological richness is more strongly associated with indoor than outdoor activities during the Pandemic. The regression coefficients suggested that indoor rather than outdoor activities were more strongly correlated with psychological richness, suggesting that an increase in new activities at home might improve psychological richness. Although speculative, these findings indicate the possibility that behavioral changes differentially affected different well-being dimensions.

### 4.2. Limitations and future directions

The present study has several limitations that should be considered when interpreting the results. The first limitation is that the study lacked a longitudinal assessment of well-being changes. The present study examined the relationship between behavioral changes before and after the onset of the Covid-19 pandemic and the “current” well-being because it was challenging to assess past subjective well-being reliably. Secondly, the causal relationship between behavioral changes and well-being remains unclear. We discussed the results of the study by assuming behavioral changes affected well-being. However, it might be possible that the initial well-being level before the Pandemic affected the quality and quantity of behavioral changes, such that people with a high level of well-being before the Pandemic tended to increase the frequency and/or the variety of alternative activities during the Pandemic. Thirdly, the present study posits intentional behavioral changes as increased or decreased activities due to the COVID-19 pandemic. However, the motivation behind these behavioral changes was not assessed. Therefore, future research should go beyond behavioral changes and assess whether behavioral changes were indeed “intentional” or influenced by external factors such as invitations or commitments. Fourth, we measured the behavioral changes based on participants’ recall rather than direct observations, which might have introduced some measurement errors. Fifth, the present study did not consider individual differences, such as differences in the level of social support and the region of residence. For example, social support may be associated differently with different types of well-being (Siedlecki et al., 2014), so the impact of individual differences in social support is also worthy to be investigated. Note that, regarding the regional differences, we performed an additional analysis using 547 participants living in densely populated prefectures (more than 1,200 people/km^2^) and found comparable results with the main analysis. In terms of transport, however, there was no significant association between use of private vehicles and well-being, which may be caused by a low possession rate of private vehicle in city areas.

The association between behavioral changes and well-being found in the present study should be attributed to the unique effect related to infections such as COVID-19, because the behavioral-change items were selected depending on the correlation with coping behaviors to COVID-19. Nevertheless, it is possible that the findings in the present study (i.e., the maintenance of well-being by behavioral changes) can be generalized beyond the COVID-19 pandemic, such as lifestyle changes due to restrictions associated with disasters, child rearing, or physical injuries. Thus, it is worthy to examine the influence of behavioral changes due to these factors on well-being in the future studies.

## 5. Conclusion

The present study demonstrated that individuals whose leisure activities (e.g., traveling and shopping), transport modes (e.g., public transport), and working environment (e.g., working at office) were unintentionally restricted by the COVID-19 pandemic have reduced their current well-being. The present study also demonstrated that individuals could improve their well-being by engaging in alternative leisure activities (e.g., exercising at home and online shopping), transport modes (e.g., private car), and working environment (e.g., working from home) even when travel and outdoor activities are restricted during Pandemics. The findings of the present study suggest that people can maintain or improve their well-being through intentional increases in alternative activities even though external factors, including lockdowns, reduce people’s well-being during pandemics. The present study would provide valuable insights into what behavioral modifications would be beneficial for preserving one’s well-being when confronted with comparable social or political constraints in the future.

## Data availability statement

The original contributions presented in the study are included in the article/Supplementary material, further inquiries can be directed to the corresponding author.

## Ethics statement

Ethical review and approval was not required for the study on human participants in accordance with the local legislation and institutional requirements. The patients/participants provided their written informed consent to participate in this study.

## Author contributions

NK, MK, and YT developed the study concept and design and wrote the manuscript. NK collected and analyzed the data. All authors contributed to the article and approved the submitted version.

## Conflict of interest

The authors declare that the research was conducted in the absence of any commercial or financial relationships that could be construed as a potential conflict of interest.

## Publisher’s note

All claims expressed in this article are solely those of the authors and do not necessarily represent those of their affiliated organizations, or those of the publisher, the editors and the reviewers. Any product that may be evaluated in this article, or claim that may be made by its manufacturer, is not guaranteed or endorsed by the publisher.
