# Supplementary material for: Association between intentional behavioral changes and well-being during the COVID-19 pandemic
Source: Front Psychol. 2023 Jul 13;14:1201770. doi: 10.3389/fpsyg.2023.1201770 (PMC10373062; doi:10.3389/fpsyg.2023.1201770)
Supplement: Supplementary file 2 [file Table_2.DOCX]

Appendix

Table S1. Results of multiple regression analyses including three types of target predictors, in which the significant variables observed in the separately conducted analyses were used.

|  | β | *t* | 95% CI |
| --- | --- | --- | --- |
| **Hedonic well-being** |  |  |  |
| (Intercept) | 3.90 | 49.91 | [3.85, 3.95] |
| gender (0; male, 1; female) | -0.12 | -1.49 | [-0.17, -0.07] |
| age | -0.15*** | -3.66 | [-0.18, -0.12] |
| marriage (0; unmarried. 1; married) | -0.39*** | -4.33 | [-0.45, -0.33] |
| income | 0.26*** | 5.88 | [0.23, 0.29] |
| indoor activity | 0.08^†^ | 1.87 | [0.05, 0.10] |
| outdoor activity | 0.10* | 2.18 | [0.07, 0.13] |
| private vehicles (driver) | 0.12** | 2.86 | [0.09, 0.14] |
| public transport (buses, trains) | 0.08^†^ | 1.86 | [0.05, 0.11] |
| control of homeworking | 0.16*** | 3.85 | [0.13, 0.19] |
| work at office | 0.04 | 0.92 | [0.01, 0.07] |
| **Eudaimonic well-being** |  |  |  |
| (Intercept) | 3.82 | 56.16 | [3.77, 3.87] |
| gender (0; male, 1; female) | 0.08 | 1.16 | [0.03, 0.13] |
| age | -0.11** | -3.12 | [-0.14, -0.09] |
| marriage (0; unmarried. 1; married) | -0.27*** | -3.47 | [-0.33, -0.22] |
| income | 0.20*** | 5.16 | [0.17, 0.22] |
| indoor activity | 0.10** | 2.87 | [0.08, 0.12] |
| outdoor activity | 0.07^†^ | 1.84 | [0.05, 0.1] |
| private vehicles (driver) | 0.10** | 2.85 | [0.08, 0.12] |
| public transport (buses, trains) | 0.11** | 2.78 | [0.08, 0.13] |
| work at office | -0.01 | -0.30 | [-0.04, 0.01] |
| **Psychological richness** |  |  |  |
| (Intercept) | 4.10 | 68.92 | [4.06, 4.14] |
| gender (0; male, 1; female) | -0.22*** | -3.56 | [-0.26, -0.18] |
| age | -0.02 | -0.68 | [-0.04, 0.00] |
| marriage (0; unmarried. 1; married) | -0.14* | -2.03 | [-0.19, -0.09] |
| income | 0.16*** | 4.79 | [0.14, 0.18] |
| indoor activity | 0.11*** | 3.38 | [0.08, 0.13] |
| outdoor activity | 0.05 | 1.52 | [0.03, 0.07] |
| private vehicles (driver) | 0.05 | 1.54 | [0.03, 0.07] |
| cycling | -0.05^†^ | -1.77 | [-0.08, -0.03] |
| public transport (buses, trains) | 0.09** | 2.60 | [0.06, 0.11] |
| control of homeworking | 0.13*** | 4.07 | [0.10, 0.15] |

Note. β and CI indicate the standardized regression weights and the confidence interval, respectively. "indoor activity" indicates an increase or decrease in leisure activity at home, while "outdoor activity" indicates an increase or decrease in leisure activity that involves going out. "private vehicles (driver)", and "cycling", while “public transport (buses, trains)” represents the increase or decrease in travel by public transport. "work at office" indicates an increase or decrease in work outside of the home. “Hedonic well-being” and “Eudaimonic well-being” were measured by the Satisfaction with Life Scale and the Meaning in Life Questionnaire, respectively;
*** indicates *p* < .001; ** indicates *p* < .01; * indicates *p* < .05, ^†^indicates *p* < .10.
